# Electrochemical Detection of NO and Ca^2+^ during Cold Atmospheric Plasma Treatment of Acute Wounds: Sensor Selectivity and Stability in the Plasma-Bio-System

**DOI:** 10.1007/s11090-025-10621-9

**Published:** 2025-12-12

**Authors:** Jonathan E. Thomas, Kristina Pattison, Suneel Kumar, Gagana Karkada, Duncan Trosan, Aunic Goodin, Jason Rainone, Dnyaneshwari Rananavare, Vandana Miller, Francois Berthiaume, Katharina Stapelmann

**Affiliations:** 1 Department of Nuclear Engineering, North Carolina State University, Raleigh, NC 27695 USA; 2 Center for Molecular Virology and Gene Therapy, Institute for Molecular Medicine and Infectious Disease, Department of Microbiology and Immunology, Drexel University College of Medicine, Philadelphia, PA 19102 USA; 3 Sidney Kimmel Comprehensive Cancer Center, Thomas Jefferson University, Philadelphia, PA USA; 4 Department of Biomedical Engineering, State University of New Jersey, 599 Taylor Road, Rutgers, Piscataway, NJ 08854 USA

**Keywords:** Cold atmospheric plasma, Plasma medicine, Wound healing, Oxidative stress, Biological markers, Electrochemistry, Chronoamperometry, Electrochemical wire sensors, Calcium flux, Mitochondria nitric oxide

## Abstract

Cold atmospheric plasmas (CAP) are a versatile tool in medical applications like wound healing. Its therapeutic benefits are partially attributed to the generation of biologically active reactive oxygen and nitrogen species (RONS). Characterization of RONS, however, typically only occurs after treatment. Here we report the first real-time in situ detection of CAP-generated nitric oxide (NO), and the simultaneous detection of cellular calcium ions (Ca²⁺) release using electrochemical sensors during CAP treatment of murine wounds. In vivo, NO rose rapidly within the first minute of CAP treatment but accumulated less overall than in PBS, reflecting reactions with wound-bed targets. In situ measurements revealed nearly double the concentrations of static endpoint assays, underscoring the importance of real-time detection. Ca²⁺ signals displayed transient, burst-like increases, likely due to CAP-induced membrane permeability and as response to oxidative stress. We also investigated the sensitivity, selectivity, and stability of the graphene oxide coated NO sensors and ion-selective Ca²⁺ sensors. Interference studies showed that the NO sensor also responds to H_2_O_2_ and NO_2_^−^ yet remains most sensitive to NO. Raman microscopy revealed progressive degradation of the graphene oxide layer after only one hour of CAP exposure, drastically reducing sensor currents. Improvements in NO sensor design will enable more accurate measurements for feedback control for plasma-based wound therapies. Ca²⁺ sensors are more robust and retained full functionality after three hours and repeated use providing a reliable diagnostic for immediate biological response. The results establish real-time electrochemical sensing as a powerful approach to monitor CAP-tissue interactions.

## Introduction

Cold atmospheric plasma (CAP) has emerged as a versatile tool in biomedical applications, with antimicrobial, anti-inflammatory, and pro-regenerative effects [1] that have been exploited in areas ranging from wound healing [2, 3] to cancer therapy [4]. The beneficial effects of CAP are partially attributed to the generation of reactive oxygen and nitrogen species (RONS), which also modulate cellular signaling and tissue responses [5–7]. Among the many RONS, nitric oxide (NO) is particularly significant for wound healing due to its antimicrobial properties and its role as an endogenous messenger that promotes angiogenesis, cell migration, and tissue remodeling [8–10]. The biological effects of NO are dose dependent where moderate concentrations stimulate wound healing and enhance cell migration, and excess can result in cytotoxicity, contributing to oxidative stress, chronic inflammation, or impaired healing. Defining safe and effective CAP doses for wound healing therefore ideally requires real-time quantification of NO and related species in the wound bed.

NO detection, by conventional methods, is challenging in plasma-treated biological environments because NO has a short lifetime and is rapidly scavenged [11]. Gas-phase measurements such as laser-induced fluorescence (LIF) spectroscopy, are sensitive but impractical for in situ detection in a clinical setting [12, 13]. In liquids, electron paramagnetic resonance spectroscopy (EPR) in conjunction with spin traps has been proven difficult in biological environments [14–16]. These techniques typically require large, immobile instrumentation and are not well suited for integration into clinical environments. These limitations motivate the development of compact electrochemical sensors capable of in situ monitoring of NO during CAP treatment. Graphene-based electrodes have recently attracted attention for electrochemical NO detection under physiologically relevant conditions [17, 18].

Besides measurement of CAP analytes, real-time monitoring of cellular effects could also serve as an important parameter for device control. Changes in calcium homeostasis are among the earliest changes in cells in response to environmental stressors [19, 20]. CAP has been shown to influence intracellular calcium ion (Ca²⁺) dynamics, suggesting that monitoring extracellular Ca²⁺ could provide additional insight into plasma-induced signaling. Calcium ions are central regulators of proliferation, migration, and differentiation [21], as well as angiogenesis [22]. Tissue Ca²⁺ levels are elevated throughout the wound healing cascade [23]. Calcium-releasing wound coverings are suggested to promote healing [24]. Miniaturized electrochemical sensors now enable continuous, real-time monitoring of Ca²⁺ and may offer new opportunities for noninvasive monitoring of wound biochemistry in both preclinical and clinical settings [25].

Here we report the first real-time, simultaneous measurement of NO and extracellular Ca²⁺ release during CAP treatment of murine wounds in situ using miniaturized bio-electrochemical wire sensors. Static before-and-after and real-time NO sensor measurements are compared, showing a two-fold higher NO signal during the live measurements. We characterize sensor sensitivity, selectivity, and stability in the reactive CAP environment, showing that Ca²⁺ sensors remain stable whereas graphene-based NO sensors gradually degrade and detect a composite signal of NO, NO_2_^−^, and H_2_O_2_. Despite the limitations, our CAP treatment and sensor platform provides continuous, live readouts of key RONS generated by CAP and an immediate biological response to CAP treatment, advancing the understanding of CAP-tissue interactions and providing the basis for CAP control.

## Methods

### Experimental Setup

**Fig. 1:**
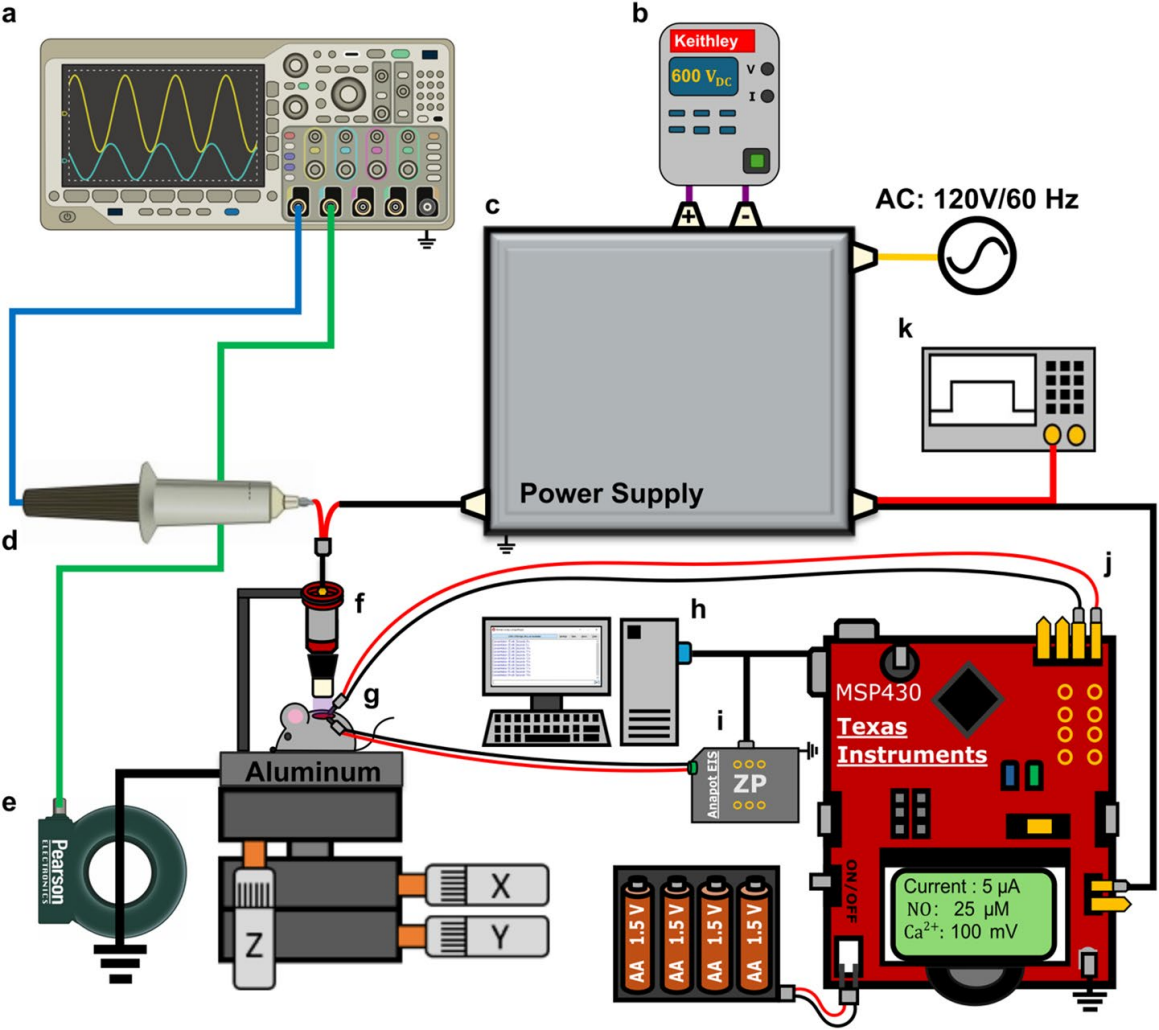
Layout of CAP delivery and measurement experimental setup. **a** Experimental setup detailing the incorporation of bio-electrochemical wire sensors to measure NO and Ca^2+^. The experimental design features an oscilloscope (Tektronix MSO64) (**a**) which helps maintain consistent voltage and current readings across various substrates. The experiment is initially powered by an external DC power supply (Keithley 2260B-800-1, 360 W) at 600 V_DC_ (**b**). The externally supplied DC voltage is then stepped up and pulsed by a µs-pulsed power supply (**c**) which is driven by a standard 120 V_AC_/60 Hz source. The CAP voltage is recorded by a high voltage probe (P6015A, Tektronix) (**d**) and its current is measured by a current transformer (Model 6585, Pearson Electronics) (**e**). The high voltage signal is delivered to a DBD electrode for targeted delivery of CAP (**f**). Once the substrates are positioned, the bio-chemical wire sensors (ZPS WIR-000–00163) (**g**), are placed on the targeted sample. All wire sensor results are interpreted via a desktop computer (**h**) and are recorded by either an Anapot EIS potentiostat (**i**) or an MCU (MSP-EXP430FR2355) (**j**). When static sensor measurements were performed a Tektronix AFG3052C function generator (**k**) served as pulsing input instead of the MCU

A volume dielectric barrier discharge (vDBD) electrode configuration was used to generate CAP, described in detail elsewhere [26]. In brief, the electrode consisted of a 7.5 mm diameter copper rod encased in a 2 mm thick aluminum oxide (Al_2_O_3_) dielectric (Kyocera 201–11010-0150; outer diameter: 10 mm, inner diameter: 8 mm, length: 15 mm) crucible. A grounded aluminum plate served as the treatment platform, on which each substrate was placed for CAP exposure. To ensure a consistent treatment distance of 1 mm, the high-voltage electrode was mounted on an XYZ-translational stage (B08 × 78HLSG, Hilitand) affixed to an aluminum plate. When substrates were treated within well plates, a custom-designed grounding sheet was affixed to the underside of each plate to enable electrical contact with the grounding platform. The electrode was driven by a custom-built microsecond-pulsed power supply, with its construction and characterization described in prior work [27, 28]. The system was powered via an external DC power supply (Keithley 2260B-800-1, 600 VDC, 360 W) and an AC power source to operate the pulsing circuit. A function generator (Tektronix AFG3052C) provided pulsed input with a fixed repetition frequency of 300 Hz and enabled control over the applied voltage through modulation of the duty cycle. During real-time data acquisition, a Texas Instruments micro-controller (MSP-EXP430FR2355) was interchangeably used to serve as pulsing input with programmable timer functionality. Current and voltage were measured using a high-voltage probe (Tektronix P6015A) and a current transformer (Pearson 6585), monitored using an oscilloscope (Tektronix MSO64) and held constant at 20 kV_pp_ and 20 mA_pp_. Under these conditions, the system maintained a delivered power of ~ 0.6 W per pulse. The same vDBD setup and electrical parameters were used across all experimental sites to ensure consistency and reproducibility. An overview of the experimental setup can be seen in Fig. 1.

### Data Acquisition

Custom designed non-enzymic wire sensors (Zimmer & Peacock AS, ZPS WIR-000–00163, Norway) were purchased with the goal of detecting and characterizing NO and Ca^2+^ during CAP treatment of biological substrates. Each sensor operated by measuring electrochemical redox reactions with a 70% platinum (Pt), 30% iridium (Ir) working electrode (WE) surface, relative to a combined silver (Ag) silver chloride (AgCl) reference electrode (RE) and counter electrode (CE). Signal generation relied on the physical adsorption of liquid samples onto an exposed WE sensing region, where target analytes underwent redox processes that produced a measurable current or voltage. Sensors were suspended in either cell-free phosphate buffered saline (PBS), or PBS placed on the mouse wound site during CAP treatment. To prevent photodegradation of the selective membrane coatings and the Ag/AgCl reference cells, sensors were stored at 3–5 °C and 30–50% relative humidity in a light-restricted storage environment between and after experiments.

Sensor data was collected either before and immediately after CAP treatment (static) or during CAP treatment (real-time in situ). For all static sensor measurements, signals were taken with the Anapot electrochemical impedance spectroscopy (EIS) potentiostat (Zimmer & Peacock, ZP1000080, Norway). Data was recorded and then interpreted through the vendor supplied software PSTrace 5.9 (PalmSens BV, the Netherlands). A custom Python script was developed to process the recorded data and extract sensor readings at defined diffusion-limited sampling intervals.

All real-time in situ sensor data was acquired with a custom designed 16-bit microcontroller (MCU) that was configured to operate as a potentiostat for potentiometric and chronoamperometric electrochemical techniques. Two 12-bit analog-to-digital converter (ADC) ports were initialized to measure voltage data (Ca^2+^), and current data (NO) simultaneously. Current data was refined with an onboard transimpedance amplifier, and all signals were processed via in-built low pass filters (cutoff: 159.15 Hz) to eliminate interference during CAP treatment. Timer functionality was enabled on the MCU to allow for selectable treatment times. Selections were made through toggle switches and a graphical user interface (GUI). Timer functionality was tied to onboard pulse width modulation (PWM) control and served as a pulsing input to cease plasma treatment once time had elapsed. Data retrieval was handled through universal asynchronous receiver/transmitter (UART) serial logging via Termite 3.9 (CompuPhase, the Netherlands). Data export and analysis were facilitated using custom Python scripts for the MCU after treatments. All programming and functionality were performed in C and Assembly coding languages and compiled via Code Composer Studio.

### Ca^2+^ Sensors and Calibration

Ca^2+^ wire sensors (Zimmer & Peacock AS, ZPS CAL-000–00285, Norway) are functionalized with an ion-selective membrane to measure extracellular calcium-ion release from biological substrates with a range of 0–10 mM. The PVC-based ion-selective membrane (ISM) incorporating Calcium Ionophore I (ETH-129) is a cation-specific carrier, forming stable complexes with metal cations, but favoring Ca²⁺ due to its coordination chemistry and ionic properties. Ca²⁺ ions from the solution selectively bind to the ionophore in the membrane, creating a charge separation at the membrane/solution interface. To measure the change in membrane potential when exposed to Ca²⁺ in the solution, open circuit potentiometry (OCP) was used where a continuous OCP trial commenced and provided real-time potentiometric data until treatment time elapsed.

**Fig. 2:**
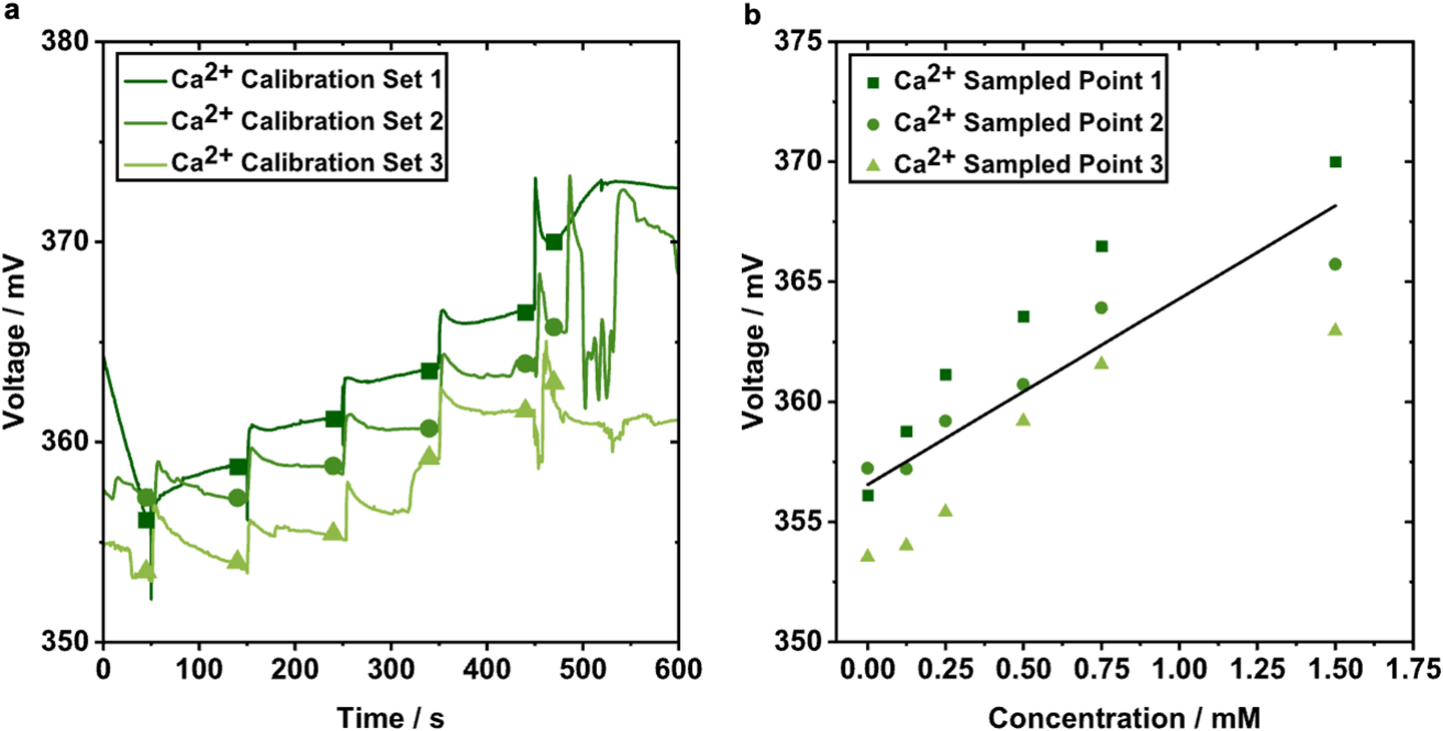
Calibration of Ca^2+^ wire sensors in PBS. **a** Measured voltage response curves correlating to serial dilutions of 0, 0.125, 0.25, 0.5, 0.75, and 1.5 mM Ca^2+^ pipetted into 500 µL of PBS. Each step correlates to an increase of Ca^2+^ in the PBS. **b** Standard curve (black line) established based on linear relationship of sampled data points (dark green, square; green, circle; light green, triangle) at each plateau of the step-function

To calibrate each Ca²⁺ sensor, solutions of known concentration were prepared by combining calcium nitrate tetrahydrate (Ca(NO_3_)_2_·4H_2_O as the detection solution and sodium chloride (NaCl) as the background electrolyte to stabilize ionic strength. 50 mL of a 10 mM stock solution of the calcium calibration solution was prepared in PBS. An intermediate stock solution was created by adding 3 mL of the 10 mM stock solution to 7 mL of PBS to create a 3 mM solution. This intermediate solution was then used to create solutions with final concentrations of 0, 0.125, 0.25, 0.5, 0.75, and 1.5 mM in 500 µL PBS. Sensors were then tested in each respective calibration solution to confirm a detectable response. Sensor calibration was also performed by stepwise additions of the 3 mM stock solution to 500 µL of PBS to achieve the final concentration of 0.125, 0.25, 0.5, 0.75, and 1.5 mM, respectively. This produced a stepwise voltage response characteristic of Ca²⁺ activity (Fig. 2a), from which a linear standard curve was generated (Fig. 2b). Following each Ca²⁺ addition sampling points on the voltage response curve were taken from the plateau (quasi-steady-state) regions of each concentration step in Fig. 2a. Using the Nernst equation [29], the observed voltage response was approximately 10 mV per 1m M increase in Ca²⁺ concentration.

### NO Sensors

To detect nitric oxide (NO), NO-selective wire sensors (Zimmer & Peacock AS, ZPS NIO-000–00261, Norway) with a detection range of 0–200 µM were sourced. The NO sensor utilizes a graphene oxide (GO) layer electrodeposited onto a carbon substrate. The graphene coating combined high electrical conductivity with surface chemistry to enhance NO selectivity. Its non-covalent modifications preserved its sp2 carbon structure, to maintain high charge carrier mobility and enabled selective binding through π–π stacking, while covalent attachments offered anchor points for catalytic molecules to facilitate the oxidation of NO [17]. An optimal oxidation potential for NO detection in PBS was determined through cyclic voltammetry (CV). The measured CV oxidation peak revealed an ideal operating voltage (*E*_dc_) of 1.05 V_DC_ (Pt/Ir vs. Ag/AgCl) for the necessary electrochemical reaction of NO → NO^+^ + e^−^ to occur. To measure NO, chronoamperometry was employed by applying a constant potential of 1.05 V_DC_ and recording the resulting current due to electron transfer. For static sensor measurements, a recording rate of 0.1 s was used, with a selected current range of 1 nA to 1 mA and a starting current of 100 µA. Based on the geometric surface area of the working electrode (1 mm²), and the diffusion coefficient of NO in aqueous solutions (2.21 ± 0.04 × 10^− 5^ cm^2^ s^− 1^) [30], the estimated diffusion time to the electrode surface was 15 s. To enable repeated, time-resolved measurements during continuous CAP exposure in situ, pulsed chronoamperometry was implemented. In this method, the sensor was pulsed at discrete 30 s intervals which allowed the electrode surface to reset between measurements and reinitiate the NO oxidation reaction. This measurement methodology minimized fouling, reduced background measurement drifts, and enabled repeated NO quantification in real-time. NO sensors were factory calibrated at Zimmer & Peacock AS (Norway).

### NO Detection in PBS

NO sensors were initially tested in PBS for static and real-time measurements. These tests could not be performed for Ca²⁺ sensors, as CAP treatment alone would not generate Ca²⁺ in the absence of cellular or tissue sources.

For static measurements, 500 µL of calcium- and magnesium-free PBS (Corning 21–040-CV) was plated into individual wells of a 24-well plate. Wells were treated for 60, 120, 180, 240, or 300 s. Prior to treatment, sensors were submerged in PBS to obtain a baseline reading for 120 s. Immediately following CAP exposure, sensors were again submerged into the treated PBS, and NO measurements were recorded for another 120 s using the AnaPot EIS potentiostat. For static NO measurements, a 20 s sampling interval was chosen to allow the diffusion field to stabilize under constant bias. After each trial, sensors were removed, rinsed with biosensor rinse solution (ZPCH 900-000-00455), and thoroughly dried. A 60 s recovery interval was applied between measurements to prevent potential interference from residual RONS generated in prior treatments. Each experiment was performed in triplicate.

For real-time measurements, sensors were submerged in 500 µL of PBS on a microscope slide horizontally by minimally disrupting the surface tension of the liquid on the slide. The sensor being used was then selected on the MCU, and a baseline reading was conducted for 30 s prior to treatment. Following each baseline reading, a desired treatment time (60, 180, or 300 s) was selected via the MCU. CAP treatment was carried out autonomously and continued until the treatment time had elapsed. Sensor readings were recorded continuously during CAP treatment and onboard MCU diagnostics processed current and voltage readings into concentration values within 10 ms. Readings were sampled every 0.1 s until the selected treatment time elapsed. Sampled pulse-chronoamperometric data points corresponding to NO concentration were taken at the midpoint (15 s) of each 30 s pulse period, corresponding to the diffusion-limited steady-state portion of the current response.

### Selectivity of the NO Sensor

To validate the accuracy of NO sensor measurements, selectivity tests were conducted to ensure that meaningful comparisons could be made between static and real-time recordings. Achieving selectivity for NO is particularly challenging in electrochemistry, as the oxidation and reduction potentials employed (>0.8 V vs. Ag/AgCl) often overlap with those of other electroactive species [31]. This is further compounded by the high reactivity of NO itself, which undergoes rapid oxidation to nitrite (NO₂⁻) under physiological conditions [32]. CAP treatment further complicates this scenario by generating a wide spectrum of RONS, many of which may be detected concurrently during NO measurements. Among these, hydrogen peroxide (H₂O₂) and nitrite (NO₂⁻) represent the most likely interferents at the platinum/iridium working electrode biased at 1.05 V vs. Ag/AgCl. Both species are electroactive at this potential as H₂O₂ is known to oxidize at potentials as low as 0.5 V [33], while NO₂⁻ is readily oxidized at 0.7 V [34].

To assess the selectivity of these NO sensors, a series of post-CAP exposure experiments were designed. As NO is very short-lived, there should not be a measurable signal after treatment. Following the established protocol for NO detection in PBS, individual wells of a 24-well plate were exposed to CAP for 60, 180, and 300 s. NO sensor readings were then collected at post-treatment intervals of 5, 10, 15, 30, 60 min, as well as at 24 h. For each timepoint, sensors were immersed in CAP- treated PBS for 120 s, and measurements were taken with the AnaPot EIS potentiostat. To further characterize the reactive species produced alongside the NO measurements, colorimetric assays were performed on the CAP-treated PBS to quantify the amount of H_2_O_2_ and NO_2_^−^ concentrations produced in the solutions, both potentially interfering with the measured NO signal. Colorimetric assays were carried out on 500 µL of PBS that had been exposed to CAP for each timepoint. Samples were subsequently transferred into 96-well plates and processed according to manufacturer protocols for a nitrite detection kit (Spectroquant, Supelco, 1.14776.0001) and a hydrogen peroxide detection kit (Spectroquant, Supelco, 1.18789.001). Lastly, a standard curve to determine concentration values was obtained by using optical absorption spectroscopy (OAS) via measurements from a microplate reader (BioTek 800 TS Absorbance Reader).

### Sensor Durability Measurements

To assess long-term operational stability, NO and Ca^2+^ sensors underwent long-term CAP exposure testing. Following CAP exposure, each sensor was subjected to repeated electrochemical measurements to evaluate changes in either current or voltage responses over time compared to an untreated control sensor. Decreases in current or voltage were monitored as indicators of sensor degradation or loss of sensitivity due to prolonged plasma interaction. In parallel, morphological changes to the sensor surface were analyzed using Raman microscopy to assess structural integrity of the permselective sensing materials.

Three electrodes of varying exposure were used for this study, a pristine never used electrode, a lightly used (1-hour of CAP exposure), and a heavily used electrode (3-hours of CAP exposure). To detect morphological changes, a confocal Raman microscope (Senterra II, Bruker Corporation) equipped with a 20 mW laser at a wavelength of 532 nm was used. The microscopy images were taken with a 20x objective lens (Olympus MPlan 20x) while the spectra were taken with a 50x objective lens (OLYMPUS). Data was recorded between 0 and 3700 cm^− 1^. For each electrode, 16 spectra acquisitions were taken at different locations on the electrode using the OPUS software (Bruker Version 7.8). Each spectrum was baseline corrected using piecewise polynomial interpolation through user-selected baseline anchor points. The spectra were then normalized using a simple min-max normalization. Spectra were then averaged and the standard deviation between each of the 16 spectra was recorded. Data post-processing was performed in Matlab. 

### Full-Thickness Wound Measurement

A full thickness mouse wound model was created as described previously [26]. A batch of 9 adult male mice (BALB/c, 9-week-old) were purchased (Charles River Laboratory, Wilmington, MA, USA) and acclimatized for a week to standard laboratory conditions (4–5 mice/cage, 72 ± 2 °F, 30–70% humidity, 12 light :12 dark cycle) before surgery. All mice were provided with a standard diet (PMI Nutrition International, Brentwood, MP) and water *ad libitum*. A day before surgery, the dorsal region was shaved using an electric clipper and Nair™ cream under isoflurane anesthesia (Henry Schein, USA) and then mice were returned to their individual cages. On the day of surgery, mice were similarly anesthetized. To prevent eye dehydration during the experiment, artificial tear cream was applied to the corneas. For pain management post-surgery, Ethiqa XR (3.25 mg/kg) was subcutaneously injected. For wound induction, the wound site was disinfected with Betadine and 70% ethanol three times alternatively. A 10 mm diameter full-thickness excisional wound was created on the dorsum of each mouse using a biopsy punch.

Following wounding, 50 µL of PBS was applied to the wound, and sensors were submerged horizontally on either side of the wound bed for a short period. Baseline readings for both NO and Ca^2+^ were initiated from the GUI of the MCU and lasted approximately 30 s. Once baseline readings were recorded, a corresponding treatment time (60, 180, 300 s) was selected from the MCU. Once confirmed, CAP treatment was carried out autonomously and continued until the treatment time had elapsed. Sensor readings were recorded continuously during CAP treatment and onboard MCU diagnostics processed current and voltage readings into concentration values within 10 ms during treatment. Readings were sampled every 0.1 s until the selected treatment time elapsed. Immediately after treatment, sensors were removed, wound sites were photographed and covered with Tegaderm™ (3 M) to protect the wound environment.

### Statistical Analysis

Statistical analysis was performed in OriginPro 2025 (OriginLab Corporation, USA). One-way ANOVA with Tukey’s post-hoc test was used, unless stated otherwise. p-values < 0.05 were considered statistically significant (*p < 0.05, **p < 0.01, ***p < 0.001, and ****p < 0.0001). All bar graph results are presented as mean ± SEM, unless otherwise stated.

## Results

### NO Sensor Measurements in CAP Treated PBS

**Fig. 3:**
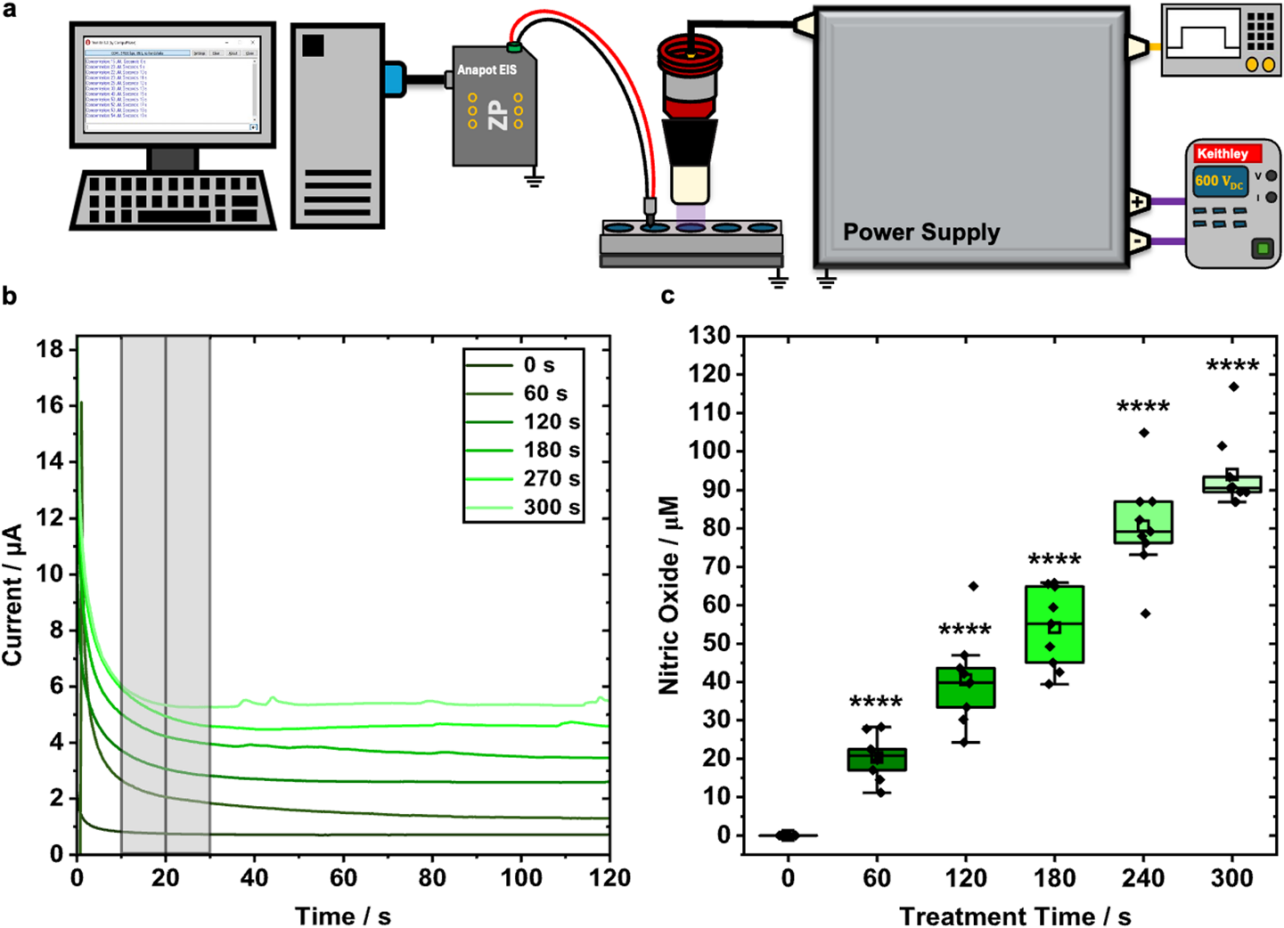
Static measurement of NO in PBS. **a** Experimental setup for treating PBS with CAP and measuring nitric oxide. **b** Current response curve produced for NO measurements, performed by wire sensors within PBS prior (0 s) and directly after CAP treatment (60 s, 120 s, 180 s, 240 s, and 300 s). Sampling time for each response curve is indicated by the grey region centered at 20 s. **c** Concentration values of NO corresponding to CAP treatment doses in panel b. Nine data points (black diamonds) are plotted for each treatment time, representing three independent experiments performed in triplicate (*n* = 9) on the PBS for each CAP exposure duration. The statistical mean (clear, squares), median (black, line in the center of box plot), interquartile ranges (box range: 25th – 75th percentile of data set), and outer legs of each box (lower 5th percentile and upper 95th percentile of data set) are showcased for each dataset. Outliers were shown above or below the outer legs. Statistical differences between 0 s and treatment groups are identified by: ****p < 0.0001

Sensor performance was first evaluated in 500 µL of cell-free PBS in a 24-well plate (Fig. 3a). Static sensor measurements were taken before and immediately after each treatment. Current readings before treatment were near 0 µA (Fig. 3b). After CAP exposure, the current response curves shifted up and plateaued at higher current values, reflecting higher NO levels at the 20 s sampling point. After 60 s of CAP treatment, NO concentrations rose by an average of 20 µM and continued to increase in a dose-dependent manner with longer exposures (Fig. 3c). Variability between trials decreased at longer treatment times, showcasing a more consistent response from the sensors in the PBS to CAP. All treatment times produced statistically significant increase relative to baseline.

**Fig. 4:**
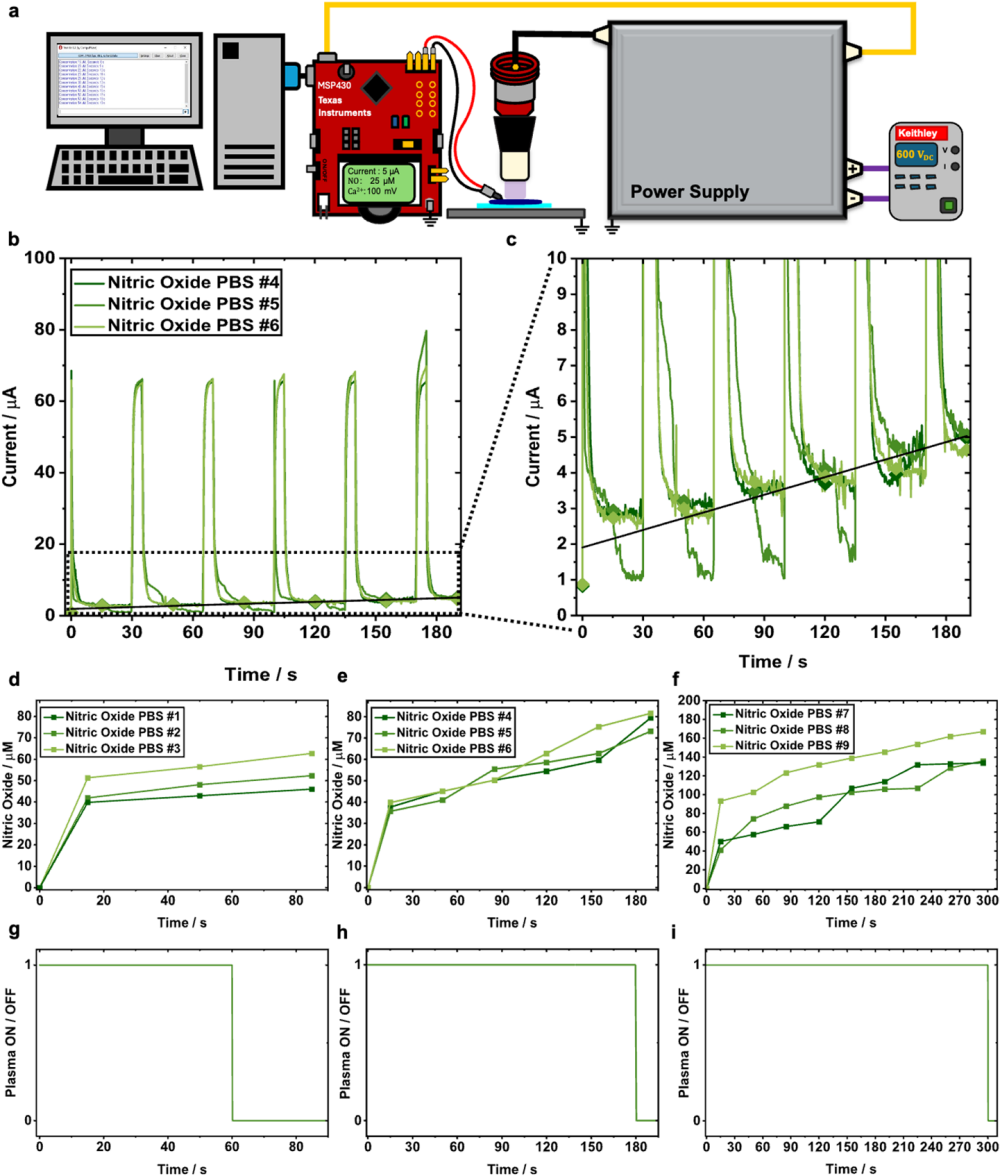
Challenging the controller in situ for real-time detection of NO. **a** Experimental setup for performing real-time detection of NO. **b** Live pulsed chronoamperometry current response curves during CAP treatment of PBS for 180 s. **c** Live current response curves zoomed in from (**b**) to better visualize slope increase over 180 s. Sampled data points on the response curves were used to determine NO concentration at the given timestamps in the PBS solution. **d-f**, NO concentration detected in each well of PBS, derived from the current curves depicted in panel **b** and **c**. **g-i** PWM signal corresponding to each treatment in panel d-f. The CAP device stays ON (1) until the set treatment time had elapsed and, the CAP device turns OFF (0)

To monitor NO generation in real time, the sensor was then positioned horizontally in 500 µL of cell-free PBS on a microscope slide to best expose the sensor to CAP treatment (Fig. 4a). The current response curves for the real-time in situ pulsed chronoamperometry experiments are displayed for the 180 s treatments as an example in Fig. 4b with a magnification of the plateauing signal used for the concentration determination displayed in Fig. 4c. NO sensors were pulsed at a pulse period (30 s) that would enable detection in accordance with the calculated diffusion limited response time of the NO in PBS (15 s). The ideal NO concentration sampling regime was determined to be the midpoint of each current response curve. By sampling at the midpoint, measurements avoided both the initial capacitive charging artifacts (first 14 s) and the later-time signal decay (last 16 s), providing a more stable and reproducible estimate of NO concentration. Measurements continued to be sampled in this fashion until the allocated treatment time expired. For all treatment times (60 s, 180 s, and 300 s) a dose-dependent increase in NO is observed (Fig. 4d-f), similar to the static sensor experiments (cf. Figure 3). Each curve displays a sharp rise in NO concentration within the first 15 s of CAP treatment, consistent with a rapid NO flux to the electrode surface followed by a diffusion-limited steady state, similar to observations in our previous study with a H_2_O_2_ sensor [26] and other reports [35–37].

Notably, real-time measurements yielded nearly double the NO obtained with the static method by 60 s. This likely reflects NO loss during sample handling in the static approach, given its short lifetime in oxygenated aqueous media. Once treatment times had elapsed, the plasma device automatically shut off and logged total exposure time (Fig. 4g-i), as introduced in our previous work where the plasma treatment was shown to automatically terminate either when a set treatment time or a set concentration of one of the measured analytes was reached [26].

### In Vivo Sensor Measurements

**Fig. 5:**
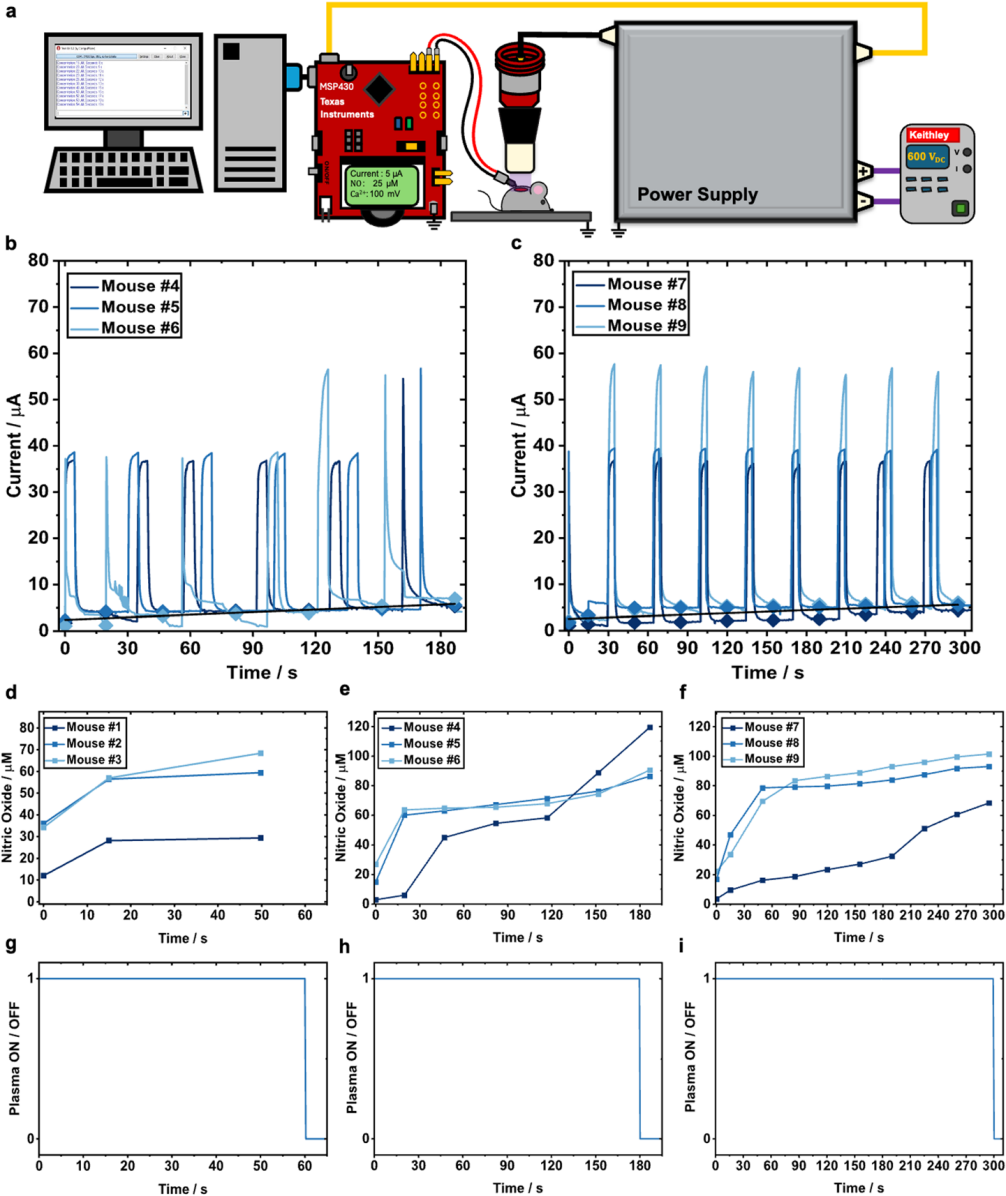
In vivo real-time measurement of NO in situ at suboptimal, optimal, and excessive CAP doses. **a** Experimental setup for in vivo dose definition study. **b** Live pulsed chronoamperometry current responses in situ during treatment of mice for 180 s. Sampled data points on the response curves were used to determine NO concentration at the given timestamp on the wound of mice 4–6. **c** Live pulsed chronoamperometry current responses in situ during treatment of mice for 300 s. Sampled data points on the response curves were used to determine NO concentration at the given timestamp on the wound of mice 7–9. **d-f** NO concentration detected in each mouse for 60 s, 180 s, and 300 s, derived from the current response curves. **g-i** PWM signal corresponding to each treatment in panel d-f. The CAP device stays ON (1) until the set treatment time has elapsed and, the CAP device turns OFF (0)

**Fig. 6:**
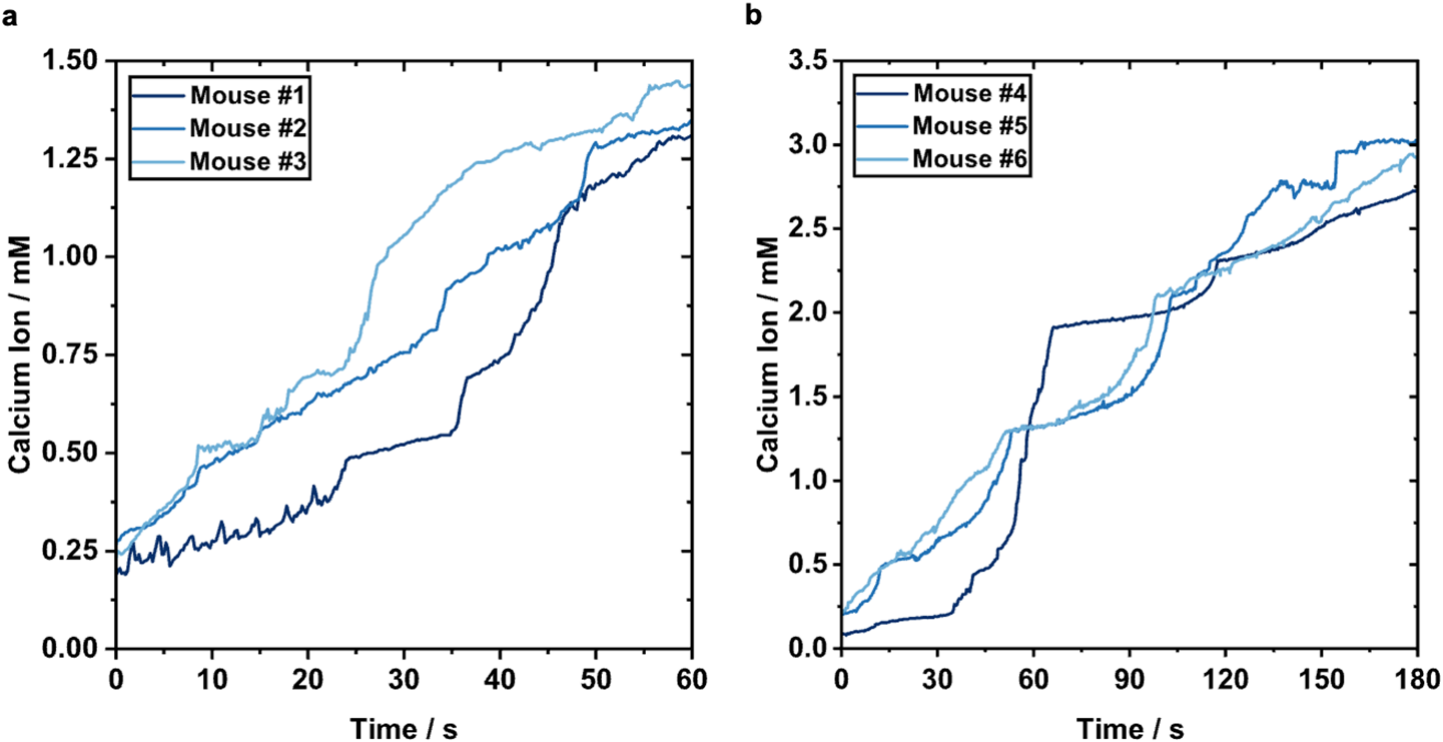
In vivo real-time measurement of Ca^2+^ in situ at suboptimal and optimal CAP doses. **a** Live open circuit potentiometry for Ca^2+^ value determination during CAP treatment of mice at 60 s. **b** Live open circuit potentiometry for Ca^2+^ value determination during CAP treatment of mice at 180 s

Baseline NO (Fig. 5) and Ca^2+^ (Fig. 6) levels were recorded in situ on the wound bed immediately after wounding (day 0). Real-time sensor measurements were then acquired during CAP treatment of wounds at suboptimal (60 s), optimal (180 s), and excessive (300 s) doses for wound healing as established in our previous research [26]. CAP treatment automatically terminated at the preset duration (Fig. 5g-i).

Current and voltage traces revealed dose-dependent increases for both analytes (NO: Fig. 5b-c, Ca^2+^: Fig. 6a-b). For NO, a biphasic response was observed during CAP exposure, characterized by a rapid initial increase within the first 10–20 s followed by a slower, diffusion-limited rise over the remainder of the treatment period (Fig. 5d–f), consistent with the behavior also seen in PBS (Fig. 4d–f). When comparing in vivo measurements (Fig. 5d–f) to those in cell-free PBS (Fig. 4d–f), the NO signal in wounds begins at a non-zero baseline concentration due to endogenous NO present in the tissue. However, the increase of NO during CAP exposure is smaller than in PBS, likely reflecting NO consumption by biological components in the wound environment. After the initial steep increase in NO signal, the rate of increase slowed, resulting in lower final concentration values especially at 300 s (101.4 µM, 93 µM, 68.4 µM) compared to in PBS (167 µM, 135.79 µM, 133.7 µM). The lower final concentrations suggest reactions with proteins (especially heme containing proteins), oxygen, and oxyhemoglobin in the wound environment, as well as detoxification through secondary reactions, for example by CAP-induced or endogenous superoxide. In our previous study, we showed that mitochondrial superoxide production increased after CAP treatment in response to the oxidative stress imposed by CAP [26]. This corroborates our findings of higher NO levels in real-time in situ PBS measurements versus in vivo within the wound bed. The spread in values is also indicative of the natural biological variability among individual mice.

Real-time in situ Ca^2+^ sensing during CAP treatment showed a time-dependent accumulation of extracellular Ca²⁺ in the wound bed for both treatments of 60 s (Fig. 6a) and 180 s (Fig. 6b), characterized by transient, step-like bursts. These rapid changes may indicate transient membrane permeability or localized intracellular Ca²⁺ efflux triggered by CAP-induced oxidative stress [38]. The Ca^2+^ signal thus reflects direct cellular responses to CAP and provides a real-time readout of plasma-tissue interaction during CAP exposure.

### Selectivity of the NO Sensor

CAP generates a wide range of RONS, including peroxynitrite (ONOO^−^), superoxide (O_2_^−^), nitrite (NO_2_^−^), nitrate (NO_3_^−^), and hydrogen peroxide (H_2_O_2_). Because many of these species are electroactive, potential interference with NO detection was evaluated. The graphene oxide (GO) functional layer on the NO sensor provides partial selectivity as its negatively charged carboxylate groups in PBS form a lamellar barrier that repel anions and favor neutral or cationic species [39]. Small, highly mobile anions such as NO₂⁻ can still penetrate the GO film to reach the electrode surface when immersed in aqueous solutions [40] and warrants further investigation. O_2_^−^ is reduced only at negative potentials (–0.2 to − 0.3 V vs. Ag/AgCl) [41] and is inactive at the 1.05 V operating bias. ONOO^−^ undergoes redox reactions mainly below 0.3 V [42] and decomposes to NO_3_^−^ and NO_2_^−^ above 1.0 V [43]. Hydroxyl radicals (·OH), while also produced by CAP, are too short-lived and require the use of a trapping probe for electrochemical detection [44–46]. NO_3_^−^ will be essentially inert at < 1.2 V and requires potentials >1.6 V for oxidation [47]. In contrast, H_2_O_2_ and NO_2_^−^ are readily oxidized at lower potentials (0.5–0.7 V) [33, 34] and are the principal interferents at a 1.05 V bias.

**Fig. 7:**
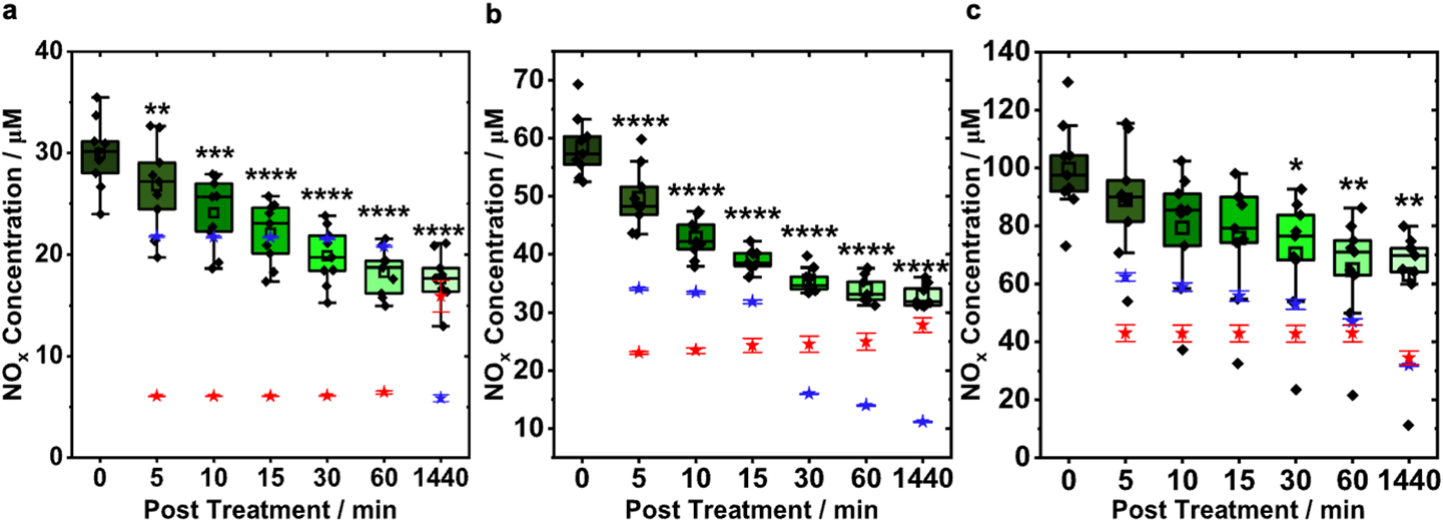
Post CAP measurement of NO analysis. Post treatment analysis of the NO signal for **a** 60 s **b** 180 s, and **c** 300 s CAP treated PBS minutes after treatment (0, 5, 10, 15, 30, 60, and 1440 min). 9 data points acquired by NO sensor measurements are plotted (black, diamond) for each treatment time, with the statistical mean (clear, squares), median (black, line in the center of box plot), interquartile ranges (box range: 25th – 75th percentile of data set), and outer legs of each box (lower 5th percentile and upper 95th percentile of data set) being showcased. Outliers were shown above or below the outer legs. All NO_2_^−^ colorimetric data (blue, star) is represented as mean ± s.e.m. (*n* = 3 per group, 6 independent experiments per treatment time). All H_2_O_2_ colorimetric data (red, star) is represented as mean ± s.e.m. (*n* = 3 per group, 6 independent experiments per treatment time). Statistical differences between 0 s and treatment groups are identified by: *p < 0.05; **p < 0.01; ***p < 0.001; ****p < 0.0001

To quantify their contribution, post-treatment static measurements and colorimetric assays for H_2_O_2_ and NO_2_^−^ were performed. Immediately after 60 s of CAP treatment, sensor readings averaged 29 µM NO (Fig. 7a), roughly half of the concentration recorded during in situ real-time monitoring (cf. Figure 4d-f). Within 5 min, the NO concentration measured by the sensors dropped to a mean value of 26.72 µM with subsequent ~ 2 µM decreases at each interval. NO_2_^−^ concentrations as determined by colorimetric assays remained near 21 µM until dropping to 5.86 µM after 24 h, coinciding with a rise in H_2_O_2_ from 6 µM to ~ 15 µM. This likely reflects the consumption of NO_2_^−^ in secondary oxidative reactions and the delayed accumulation of H_2_O_2_ formed from reactive oxygen intermediates.

These trends continued for the longer treatment times. For 180 s CAP treatments, initial NO was 58.58 µM, decreasing to 49.70 µM within 5 min and to 32.95 µM by 24 h (Fig. 7b). NO_2_^−^ and H_2_O_2_ started higher, at concentrations of ~ 34 µM and 23 µM, respectively. NO_2_^−^ concentration gradually decreased to 11.13 µM after 24 h while H_2_O_2_ continued to rise. After 300 s of CAP treatment, the NO signal reached ~ 99 µM (Fig. 7c) and then gradually decreased over the first 60 min before stabilizing at ~ 70 µM, where it remained relatively constant up to 24 h (63.2 µM). H_2_O_2_ concentrations increased from its initial value (43 µM) before slightly declining to ~ 34 µM at 24 h, while NO_2_^−^ decreased from 62.47 µM to 31.9 µM.

These results demonstrate that the sensor signal cannot be attributed solely to NO. Measurable “NO” persisted 24 h post treatment, far exceeding NO’s lifetime. Combined NO_2_^−^ and H_2_O_2_ levels post treatment roughly match the sensor’s apparent NO concentration, confirming significant interference from these CAP-generated species.

**Fig. 8:**
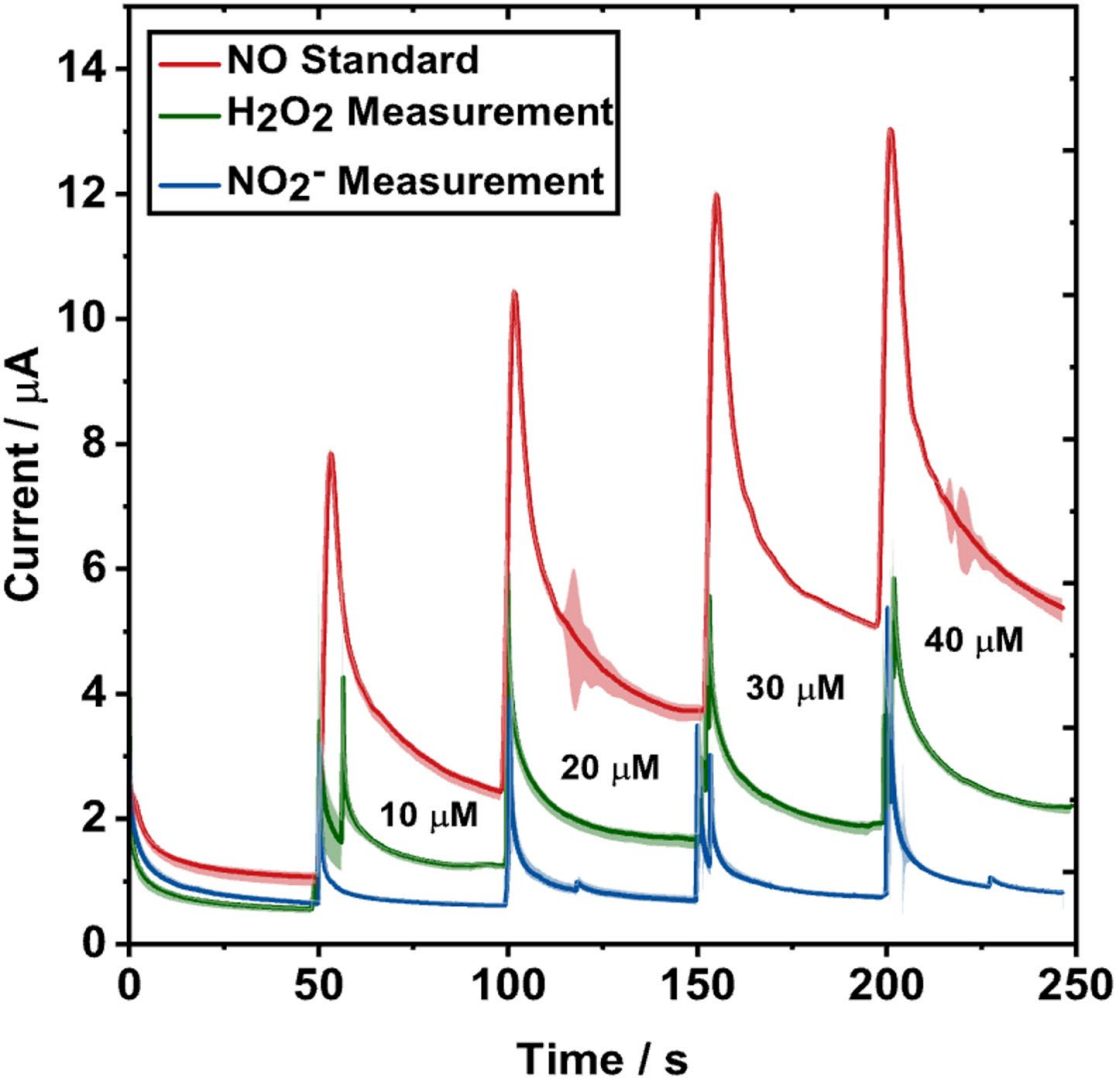
NO sensor selectivity evaluation. Hydrogen peroxide and NO_2_^−^ stock solutions were measured with the NO sensor at comparable concentration additions to determine its overall interfering current response (H_2_O_2_ measurement, green; NO_2_^−^ measurement, blue) and compared to a standard NO current response curve generated during factory calibration of the sensor at Zimmer & Peacock (NO standard, red line). While NO produces the highest signal at similar concentrations, H_2_O_2_ and NO_2_^-^ could be detected by the NO sensor

To assess whether the sensor responds preferentially to NO when it is present, the sensor output was compared in stock solutions of H_2_O_2_ and NO_2_^−^ to an NO current response curve generated during factory calibration of the wire sensor, depicted in Fig. 8. Sequential additions of 10 µM H_2_O_2_ and NO_2_^−^ produced measurable signals, but the response to NO was markedly greater. Among the interferents, sensitivity was lowest for NO_2_^−^ and intermediate for H_2_O_2_. Together with the results in Fig. 7, this data indicates that although the sensor is not fully selective towards NO, it remains most responsive to NO, supporting the estimate that approximately half of the real-time measured “NO” signal arises from actual NO contributions. Lowering the applied sensor potential could reduce the measured interference but would diminish the sensors' overall NO sensitivity, while additional permselective coatings would slow diffusion and degrade real-time response. Electropolymerized layers like poly-phenol or poly-eugenol films may help to further suppress H_2_O_2_ oxidation without sacrificing NO detection [48].

### Durability of Bio-Electrochemical Sensors in Plasma-Treated Environments

**Fig. 9:**
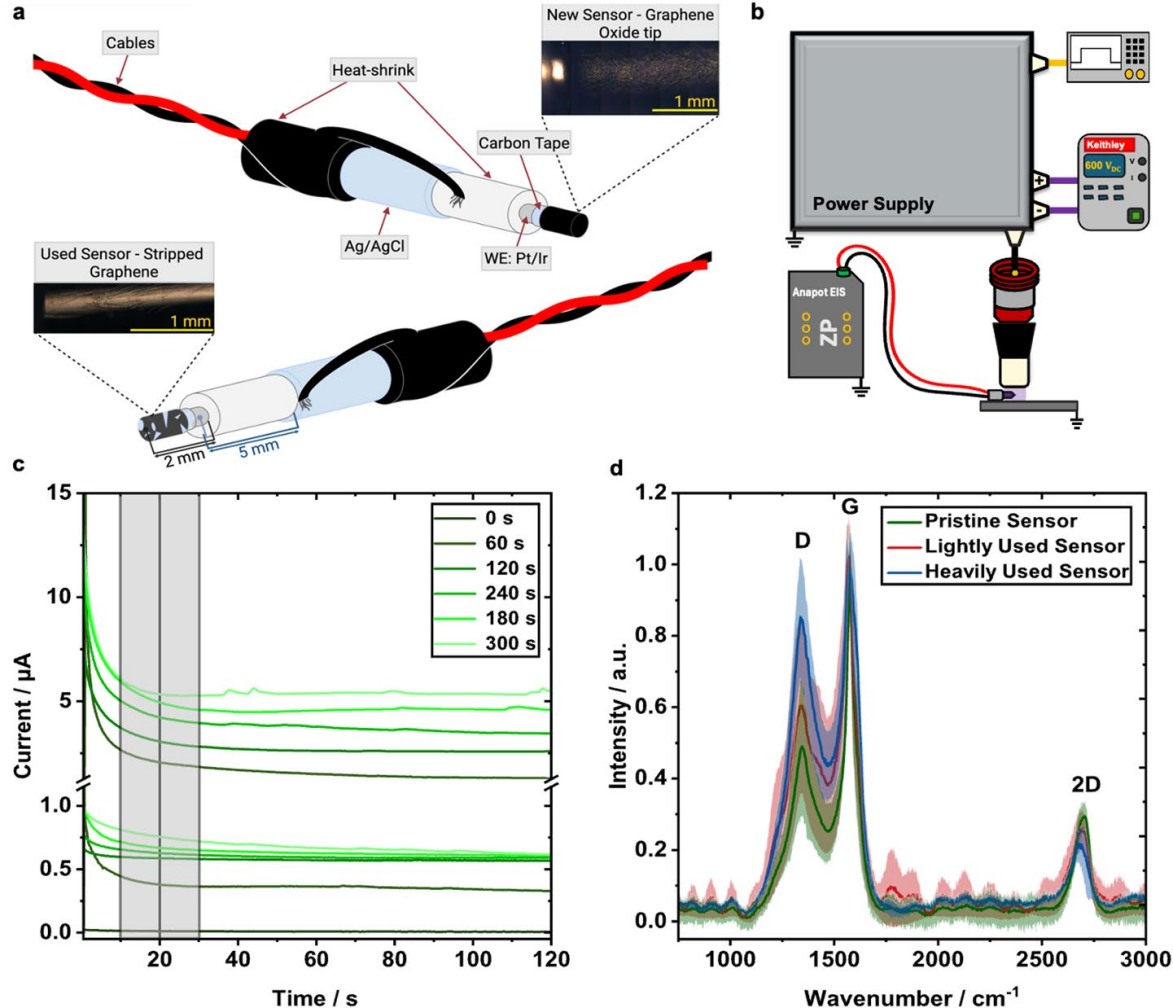
Durability Assessment of NO sensor following CAP exposure. **a** Cross-sectional layout of nitric oxide sensor before and after CAP exposure (Heavily used sensor, 3-hour CAP exposure). **b** Experimental setup for treating nitric oxide sensor at durations of 1 and 3 h. **c** NO sensor current responses of a pristine sensor (0-hour, top of graph) and a lightly used sensor (1-hour, bottom graph) for CAP treatment times of PBS (0 s, 60 s, 120 s, 180 s, 240 s and 300 s). **d** Raman spectra of NO sensors that have not been treated by CAP (pristine sensor, green line), treated for 1 h (lightly used sensor, red line), and a duration of 3 h (heavily used sensor, blue line). All Raman microscopy data was performed in triplicate with representative error bands displayed for each sensor type to reflect variability between measurements

To evaluate the sensors’ durability, sensors were exposed to CAP for 1 and 3 h and imaged by Raman microscopy. Performance was also tested by measuring current responses in CAP-treated PBS. Raman microscopy confirmed a uniform 300 µM graphene oxide layer on the pristine NO sensor (Fig. 9a). After only 1 h of CAP exposure, the NO sensor showed visible degradation of the GO surface, progressing to partial delamination and flaking by 2 h. After 3 h of CAP exposure, the electrode was nearly bare (Fig. 9a). This agrees with reports that plasma readily oxidizes GO by breaking carbon bonds and inducing lattice defects, especially at structural weak points targeted by the plasma streamers [49, 50]. The GO layer shows three prominent peaks in the Raman spectrum (Fig. 9d): G (1580 cm^− 1^), 2D (2690 cm^− 1^), and D (1350 cm^− 1^). Raman spectra of pristine, lightly used, and heavily used sensors show a rising D/G peak ratio and a declining 2D/G peak ratio with increasing exposure, indicating greater disorder and reduced thickness [51]. Broadening of the D peak suggests the formation of voids and/or grain boundaries. No noticeable change in the continuum between the D and G peaks could be observed. Functionally, CAP exposure for 1 h reduced sensor currents by an order of magnitude (Fig. 9c), reflecting severe loss of the GO layer and diminished sensitivity. Thicker or periodically reapplied GO coatings may extend sensor lifetime, but the current design is best suited for single-use applications rather than continuous clinical monitoring.

**Fig. 10:**
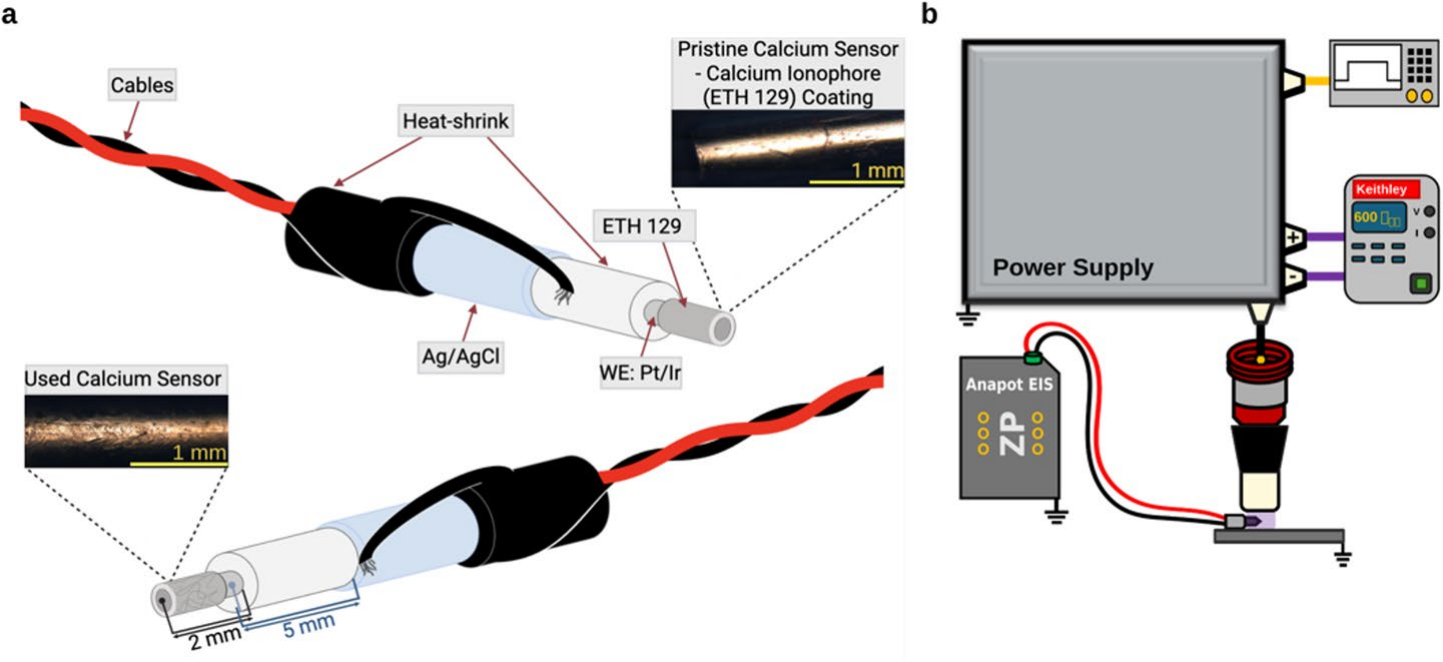
Durability Assessment of Ca^2+^ sensor following CAP exposure. **a** Cross-sectional layout of pristine calcium ion sensor before CAP exposure and microscope images of pristine and slightly used (1 h) sensors. **b** Experimental setup for treating calcium ion sensor at durations of 1–3 h

By contrast, the Ca^2+^ sensor showed greater durability and excellent stability. After 3 h of CAP exposure, no delamination, degradation, or microscopic defects were observed. Only a darkening of the ETH-129 ionophore appeared after 1 h of CAP treatment (Fig. 10), but no voltage differences were observed for the CAP treated sensors when tested with a Ca^2+^ stock solution (data not shown). CAP exposure may even enhance adhesion of the ion-selective membrane, accounting for its stability and the observed color change. Repeated use in plasma-treated biological media produced no measurable drift or morphological damage, indicating that the Ca^2+^ sensor is far more resistant to CAP than the GO-based NO sensor. Real-time Ca^2+^ sensing therefore offers a unique window into immediate biological responses to plasma, capturing CAP-induced bursts of extracellular Ca^2+^ that may reflect transient membrane permeability or oxidative stress signaling [52]. Among available analytes, Ca^2+^ measurement provides the clearest real-time indicator of tissue response during CAP treatment, whereas other biomarkers manifest only minutes to hours after treatment.

## Conclusion

In conclusion, this study demonstrates the first in situ real-time electrochemical detection of NO and Ca^2+^ during cold atmospheric plasma treatment in vivo. Our approach captured a near-linear NO increase and transient Ca^2+^ bursts during CAP treatment, that would be missed by conventionally used static end-point assays. Real-time in situ readings also consistently yielded higher NO concentrations than static before and after treatment measurements, perhaps more accurately reflecting what the biological tissue is receiving. By capturing real-time NO and Ca^2+^ responses, we have demonstrated a new method for linking CAP dose delivery with immediate biochemical signaling in wounds. Our results have highlighted both the potential and challenges associated with this approach. While Ca^2+^ sensors showed robust stability, NO sensors exhibited degradation and limited selectivity over repeated exposures. Translation to clinical use will require addressing NO accuracy, sensor reusability, and integration with safe CAP device operation. Nevertheless, the ability to monitor RONS and real-time indicators of tissue response in the form of Ca^2+^ release in real time represents an important step toward defining biologically effective CAP treatment and advancing plasma medicine toward precision therapeutic applications. Future improvements will focus on refining the NO sensor’s chronoamperometric pulse protocol to optimize NO diffusion and improve accuracy, enabling more precise dosing and potential closed-loop endpoint control. As NO and Ca^2+^ play a critical role in modulating the wound healing process from the inflammatory phase to the proliferative phase, future work will also investigate the wound healing responses in correlation to the measured analytes, providing a framework for adaptive and personalized CAP therapies with applications in wound healing and other biomedical CAP therapies.

## Data Availability

Data is provided within the manuscript or supplementary information files. The raw data that support the findings of this study are available upon reasonable request from the authors.

## Abbreviations

AC: Alternating current
ADC: Analog-to-digital converter
CAP: Cold atmospheric plasma
CE: Counter electrode
CV: Cyclic voltammetry
DC: Direct current
EIS: Electrochemical impedance spectroscopy
EPR: Electron paramagnetic resonance spectroscopy
ETH-129: Calcium ionophore I
GO: Graphene oxide
GUI: Graphical user interface
ISM: Ion-selective membrane
LIF: Laser-induced fluorescence
MCU: Microcontroller unit
OAS: Optical absorption spectroscopy
OCP: Open circuit potentiometry
PBS: Phosphate-buffered saline
PWM: Pulse width modulation
RE: Reference electrode
RONS: Reactive oxygen and nitrogen species
SEM: Standard error of the mean
UART: Universal asynchronous receiver/transmitter
VDBD: Volume dielectric barrier discharge
WE: Working electrode
E_dc_: Ideal operating voltage
